# Can Artificial Intelligence Optimize the Early Diagnosis of Invasive Candidiasis? A Systematic Review and Meta-Analysis

**DOI:** 10.3390/jof12020138

**Published:** 2026-02-13

**Authors:** Hugo Almeida, Beatriz Rodríguez-Alonso, Montserrat Alonso-Sardón, Inmaculada Izquierdo, Ángela Romero-Alegría, Virginia Velasco-Tirado, Josué Pendones Ulerio, Javier Pardo Lledías, Moncef Belhassen-García

**Affiliations:** 1 Servicio de Medicina Interna, Unidad de Enfermedades Infecciosas, Hospital Universitario de Salamanca, 37007 Salamanca, Spain; hugoalmeida6@gmail.com (H.A.); ivh.inmaizquierdo@gmail.com (I.I.); aralegria@yahoo.es (Á.R.-A.); belhassen@usal.es (M.B.-G.); 2 Centro de Investigación de Enfermedades Tropicales de la Universidad de Salamanca (CIETUS), 37007 Salamanca, Spain; sardonm@usal.es (M.A.-S.); virvela@yahoo.es (V.V.-T.); jpendones@saludcastillayleon.es (J.P.U.); 3 Instituto de Investigación Biomédica de Salamanca (IBSAL), 37007 Salamanca, Spain; 4 Área de Medicina Preventiva, Epidemiología y Salud Pública, Facultad de Medicina, Universidad de Salamanca, 37007 Salamanca, Spain; 5 Servicio de Dermatología, Hospital Universitario de Salamanca, 37007 Salamanca, Spain; 6 Servicio de Microbiología y Parasitología, Hospital Universitario de Salamanca, 37007 Salamanca, Spain; 7 Servicio de Medicina Interna, Hospital Marqués de Valdecilla, 39008 Santander, Spain; javier.pardo@scsalud.es; 8 Instituto de Investigación Valdecilla (IDIVAL), 39008 Santander, Spain

**Keywords:** *Candida*, candidemia, invasive candidiasis, machine learning, artificial intelligence, predictive models

## Abstract

The early diagnosis of invasive candidiasis remains challenging in immunocompromised and other high-risk patients, prompting interest in artificial intelligence models for assisting clinical decision-making. We conducted a PROSPERO-registered systematic review and meta-analysis of artificial intelligence-based predictive models for the early identification of invasive *Candida* infections. We searched multiple databases for studies reporting model performance in hospitalized immuno-compromised patients. Data on study characteristics, model details, validation strategy, and diagnostic accuracy were extracted. A bivariate random-effects meta-analysis was performed for candidemia prediction models with compatible data. Eight studies met inclusion criteria. Models were typically developed using retrospective hospital data with heterogeneous populations and predictors. Five candidemia studies provided threshold-based performance data for meta-analysis. Pooled sensitivity and specificity for candidemia prediction were 81.3% (95% confidence interval (CI) 72.9–87.6%) and 81.6% (95% CI 68.4–90.1%), respectively. Most models achieved high negative predictive values, whereas positive predictive values were modest, reflecting low event prevalence. The risk of bias was generally moderate to high (PROBAST), and the certainty of evidence was low (GRADE) due to study limitations and indirectness. AI models show promise for early candidemia identification with moderate diagnostic accuracy. They may be useful as decision-support tools, but further multicenter prospective validation is needed before routine clinical adoption.

## 1. Introduction

Invasive fungal infections (IFIs) are a major cause of morbidity and mortality in hospitalized and immunocompromised patients, and their epidemiology continues to evolve with changes in hematology practice, transplantation, intensive care, and antifungal exposure [1,2,3]. Within this spectrum, *Candida* species remain among the most clinically relevant pathogens, responsible for candidemia and other forms of invasive candidiasis, with substantial attributable morbidity, mortality, and healthcare burden across high-risk populations [4,5].

Despite progress in prevention and treatment strategies, the early diagnosis of invasive candidiasis remains challenging. Clinical presentations are frequently non-specific and overlap with bacterial sepsis, while diagnostic workups often occur under time pressure in critically ill patients [6]. Persistent diagnostic gaps have been highlighted across laboratory workflows for fungal diseases, including limitations in test availability, turnaround time, and interpretability in real-world settings [7]. Reviews focused on clinical practice underscore that current diagnostic pathways may be insufficient to reliably identify invasive fungal disease at an early stage, particularly when decisions about antifungal therapy must be made before definitive microbiological confirmation [8,9].

In parallel, there is continued emphasis on practical algorithms for fungal diagnosis in immunocompromised hosts and Intensive Care Units (ICU) settings, reflecting the ongoing debate between ensuring timely antifungal treatment for true infection and avoiding unnecessary antifungal exposure when candidemia is unlikely [10,11,12].

Artificial intelligence (AI) and machine learning (ML) have therefore emerged as promising tools to support earlier identification and risk stratification in infectious diseases [13,14,15]. In principle, ML models can leverage heterogeneous electronic health record (EHR) data (laboratory trajectories, physiological measurements, exposure histories, comorbidities, and microbiology-related information) to generate risk estimates at clinically meaningful timepoints. However, translation into clinical practice remains uncertain, as performance may vary markedly by population, case definition, comparator group, prediction horizon, and validation strategy.

In invasive *Candida* infections, the evidence base is expanding but remains heterogeneous. A prior systematic review of clinical predictive models for invasive *Candida* infection highlighted variability in study design, predictors, and validation, with frequent limitations affecting generalizability [16]. More recent work has explored explainable ML using automatically extracted laboratory and microbiological data for early candidemia detection [17], ML models aimed at early ICU candidemia prediction [18], and ward-based personalized ML approaches [19]. Other studies have focused on specific clinical contexts, such as malignancy-associated candidemia risk prediction [20], ICU patients with new-onset systemic inflammatory response syndrome [21], and highly selected cohorts including *Candida auris*-colonized critically ill patients [22] or cardiac assist device recipients [23]. Collectively, these studies suggest potential clinical utility but also raise concerns regarding selection bias, predictor availability at the intended “time-zero”, and limited external validation.

In light of the above, a systematic review is proposed to evaluate AI-based approaches for the early diagnosis and prediction of invasive *Candida* infections, to summarize model characteristics and performance, and to appraise risk of bias, applicability, and certainty of evidence using PROBAST- and GRADE-informed approaches. The quantitative synthesis focused on candidemia, while the narrative synthesis covered the broader spectrum of eligible *Candida*-targeted AI models across clinical settings.

## 2. Materials and Methods

### 2.1. Protocol and Reporting

This systematic literature review was conducted and reported in accordance with PRISMA 2020 recommendations [24] This systematic review was prospectively registered in PROSPERO (CRD420251269171). No amendments were made after protocol registration.

### 2.2. Research Question

To explicitly structure the diagnostic question guiding this review, we used a PICO framework. The review was designed to answer the following question: in immunocompromised adult or pediatric patients at risk of invasive *Candida* infections (P), do AI-based models (including machine learning and deep learning) developed for the early diagnosis or prediction of candidemia and/or invasive candidiasis (I), compared with conventional diagnostic strategies used in routine practice such as cultures, fungal biomarkers, clinical prediction scores, or imaging (C), accurately identify invasive *Candida* infection defined by microbiological, histopathological, or internationally accepted consensus criteria (C′)? The primary outcome was diagnostic accuracy, including sensitivity, specificity, and area under the ROC curve, with other performance metrics considered when available (O).

### 2.3. Eligibility Criteria

Inclusion criteria: We included studies evaluating AI or ML models developed for the early diagnosis, prediction, or classification of invasive *Candida* infections in immunocompromised or high-risk hospitalized populations (e.g., hematological malignancy, solid organ/HSCT recipients, prolonged neutropenia, ICU patients, or other immunosuppressive states). Target conditions of interest were candidemia and invasive candidiasis. We included both adult and pediatric populations, if applicable. Eligible studies needed to report model performance using at least one clinically interpretable metric (e.g., sensitivity and specificity, area under the ROC curve (AUC), positive/negative predictive value (PPV/NPV), or overall accuracy) and to provide sufficient methodological detail to characterize the dataset, predictors, model type, and validation approach.

Exclusion criteria: We excluded studies that were in vitro or animal experiments, studies using only synthetic or simulated datasets without direct clinical correlation, studies limited to superficial or mucosal candidiasis, and studies addressing only *Candida* colonization without an invasive infection outcome. We also excluded non-original reports and abstracts lacking full-text data.

### 2.4. Information Sources and Search Strategy

The electronic search was performed on 22 December 2025. We searched PubMed/MEDLINE (via PubMed), Scopus, Web of Science Core Collection, Embase, IEEE Xplore Digital Library, and the Cochrane Library. The search strategy combined three concept blocks: (1) AI/ML terms; (2) candidemia/invasive candidiasis terms; and (3) immunocompromised/high-risk population terms. No language restrictions were applied during the database search. Articles in languages other than English were eligible and were assessed during screening when sufficient information was available.

Full database-specific strategies are provided in Supplementary File S1. In addition, we screened reference lists of included articles to identify potentially eligible studies.

### 2.5. Selection Process

All identified records were exported to a reference manager (Rayyan^®^), and duplicates were removed. Two reviewers independently screened titles and abstracts for relevance, followed by full-text screening of potentially eligible studies. Discrepancies were resolved by discussion and consensus, with adjudication by a third reviewer if needed. To avoid double counting of patient cohorts, when multiple publications appeared to originate from the same or overlapping patient population, we included only the most comprehensive or recent report and excluded others as overlapping data.

### 2.6. Data Collection and Data Items

Two reviewers independently extracted data from each included study using a standardized form. Extracted items included the publication year, study setting and design (e.g., retrospective or prospective); patient population characteristics; target condition (candidemia vs. other invasive candidiasis) and reference standard for infection diagnosis; sample size (total number of patients or episodes, number of patients with invasive *Candida* infection, number of controls); candidate predictors and their timing relative to the prediction moment (“time-zero”); model type (algorithm) and training approach; whether external validation was performed; and reported performance metrics (e.g., AUC, sensitivity, specificity, PPV, NPV, accuracy, and calibration measures if available).

For studies eligible for meta-analysis, we additionally extracted or derived 2 × 2 contingency table data (true positives, false positives, true negatives, false negatives) at the model’s reported operating threshold, when available.

The primary outcome for quantitative synthesis was diagnostic accuracy for candidemia, specifically sensitivity and specificity at a defined decision threshold (enabling 2 × 2 contingency data). Secondary outcomes included model performance for other forms of invasive candidiasis (e.g., intra-abdominal candidiasis) and non-threshold metrics such as AUC; these were synthesized narratively when quantitative pooling was not possible.

### 2.7. Risk of Bias Assessment and Certainty of Evidence

The risk of bias for each included study was assessed using the PROBAST tool (Prediction Model Risk of Bias Assessment) across four domains: participants, predictors, outcome, and analysis. Each domain was rated as low, high, or unclear risk of bias, and overall risk of bias was determined for each study. Applicability concerns (external validity) were also evaluated in the first three domains. Two reviewers performed PROBAST assessments independently, resolving any differences by consensus.

We appraised the certainty of evidence for the primary outcome (candidemia diagnosis) using GRADE criteria adapted for diagnostic and prediction models. This involved evaluating the body of evidence for risk of bias, indirectness, inconsistency, imprecision, and potential publication bias. An initial GRADE rating was assigned (high certainty for randomized trials, which was not applicable here, or moderate for observational prediction model studies) and then downgraded based on these factors. We report the final certainty of evidence (e.g., low, very low) for the pooled diagnostic accuracy of candidemia models.

Given the limited number of studies per quantitative synthesis and the methodological limitations of publication-bias methods in diagnostic accuracy meta-analyses, we did not formally assess reporting bias/publication bias.

### 2.8. Data Synthesis and Meta-Analysis

First, a narrative/qualitative synthesis will be conducted to summarize the characteristics of all included AI models. Second, for studies with compatible threshold-based performance data, we performed a meta-analysis focusing on candidemia prediction models. Pooled sensitivity and specificity were estimated using a bivariate random-effects model (Reitsma approach) appropriate for diagnostic accuracy data. This method allowed for the simultaneous pooling of sensitivity and specificity while incorporating within-study and between-study variability. From the bivariate model, we derived a summary receiver operating characteristic (SROC) curve and calculated the summary diagnostic odds ratio (DOR) with 95% confidence intervals. Positive and negative likelihood ratios (LR+ and LR−) were computed from the pooled sensitivity and specificity. To visually present study-level results and the pooled estimates, we generated paired forest plots for sensitivity and specificity for each quantitative synthesis.

Statistical heterogeneity was explored by visual inspection of paired forest plots and the dispersion of study estimates in SROC space and was quantified using bivariate measures from the random-effects model (between-study variance components on the logit scale and bivariate I^2^, when estimable). All analyses and graphical outputs were produced using Meta-DiSc 2.0^®^ (2021). No sensitivity analyses were performed.

Due to the small number of studies, formal subgroup analyses to explore sources of heterogeneity were not feasible.

## 3. Results

### 3.1. Study Selection

**Figure 1:**
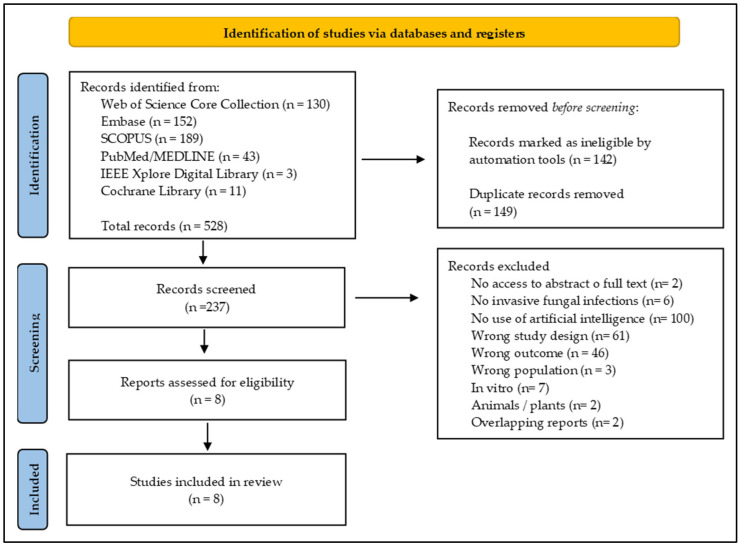
PRISMA 2020 flow diagram [24].

A total of 528 records were identified across databases. After removing 149 duplicates and excluding 142 records flagged by automation tools, 237 records were screened. Of these, 229 records were excluded (most commonly due to no AI component, inappropriate study design, or wrong outcome), leaving 8 reports assessed for eligibility. All 8 studies met the inclusion criteria and were included in the qualitative synthesis. Two additional publications were excluded as overlapping reports to avoid double counting. Two reports were excluded as overlapping publications (Yuan et al. [25] and Zhang et al. [7]) from cohorts also represented in included reports. The PRISMA flow diagram is provided in Figure 1.

### 3.2. Study Characteristics

The eight included studies were published between 2020 and 2025 and evaluated AI/ML models for the early identification of invasive *Candida* infection across diverse hospital settings and patient populations. Table 1 presents a summary of the key characteristics of these studies. Detailed information on population characteristics and the variables used for prediction can be found in Table S1. Seven of the eight studies focused on the prediction of candidemia (bloodstream infection), while one study targeted intra-abdominal candidiasis in postoperative ICU patients with intra-abdominal infections.

Study designs were predominantly retrospective (seven studies) with one prospective study (the intra-abdominal candidiasis model). Validation strategies varied: most studies used internal validation only (random splits or cross-validation, sometimes with temporally split training/test sets), and truly independent external validation was uncommon (one study). The models generally relied on routinely collected clinical and laboratory variables, often including patient exposure factors such as recent antibiotics, the presence of central lines or other invasive devices, parenteral nutrition, and other risk factors. A range of ML algorithms was represented, including tree-based ensemble methods (random forests and gradient boosting machines) and logistic regression (often with regularization).

In terms of model outcomes, most studies specifically modeled the risk of candidemia, whereas one study [26] modeled the risk of intra-abdominal candidiasis. The definition of “early diagnosis” or prediction time-zero varied across studies. The reference standard for invasive candidiasis was typically a positive culture from a normally sterile site or the fulfillment of accepted clinical criteria for proven or probable infection. Intra-abdominal candidiasis (IAC) was defined by microbiological evidence of *Candida* from intra-abdominal specimens, including *Candida* detected by direct microscopy and/or culture from purulent or necrotic intra-abdominal material obtained during surgery or by percutaneous aspiration; isolation from bile, intrabiliary duct devices, or intra-abdominal organ biopsy; isolation in secondary/tertiary peritonitis when no other pathogen was identified; or isolation from drainage tubes only if the drain had been placed <24 h before sampling [26]. The sample sizes of model development cohorts ranged from fewer than 100 patients to many thousands, with event (invasive infection) rates generally low (often <10% of the cohort, reflecting the low incidence of invasive candidiasis).

In the final dataset, eight studies were included in the narrative synthesis, covering candidemia-focused models and one study targeting intra-abdominal candidiasis. A subset of five candidemia studies reported sufficient diagnostic performance data (or allowed for reconstruction of 2 × 2 tables) and were therefore included in the quantitative synthesis (meta-analysis).

### 3.3. Risk of Bias (PROBAST) and Certainty of Evidence (GRADE)

Using the PROBAST tool, all included studies were judged to have concerning risk of bias in at least one domain, and most were rated as having overall moderate or high risk of bias. Common issues contributing to higher risk of bias were related to the Participants’ domain, and the Analysis domain (e.g., lack of calibration assessment). Several studies also had unclear timing of predictors relative to outcome (raising potential bias if predictors were ascertained post-time zero). Regarding applicability (external validity), many models had limited applicability for broader settings due to narrowly defined development populations or the use of specialized predictors unavailable in general practice.

According to GRADE criteria adapted for diagnostic models, the certainty of evidence supporting AI/ML models for candidemia diagnosis was low to very low. This was driven by the observational nature of the evidence and downgrades for risk of bias (as noted above), indirectness (since each model was developed in a specific setting that may not directly generalize to others), and, in some cases, imprecision (due to wide confidence intervals in model performance). Publication bias could not be formally assessed given the small number of studies, but we cannot exclude the possibility that only positive/interesting models were published. As a result, we have low confidence in the estimate of effect for pooled diagnostic accuracy. In brief, for the outcome of identifying candidemia, most studies’ evidence started at a “moderate” level (appropriate for prediction model studies) and was downgraded to “low” or “very low” certainty. The single study targeting intra-abdominal candidiasis was also judged as low-certainty evidence for that context (mainly due to indirectness and sample size considerations). Table 2 outlines the PROBAST risk-of-bias assessment in all domains and Table 3 defines the GRADE certainty assessment for each included study’s evidence regarding candidemia prediction.

### 3.4. Narrative Synthesis

Seven included studies developed ML models for candidemia prediction. Overall, these candidemia models were trained using routinely collected clinical and laboratory features, often in ICU or hospital ward settings, and they typically reported moderate-to-high discriminatory ability. When reported, AUC values for candidemia models were commonly in the range of ~0.81–0.92. Positive predictive values were variable (often low to moderate, reflecting the low prevalence of candidemia in many cohorts), whereas negative predictive values were generally high (often >0.95), suggesting good rule-out potential.

An explainable ML approach was evaluated using an automatically extracted dataset of clinical, laboratory, and microbiological features within the AUTO-CAND project (Italy) [17]. The model achieved a sensitivity of 0.74 and a specificity of 0.57 for early candidemia detection (AUC was not reported for the operating point used) [17]. A random forest model developed to predict candidemia in ICU cancer patients with suspected sepsis showed an AUC of 0.870, with sensitivity of 0.63 and specificity of 0.87 in an external validation cohort of 77 patients (included in our meta-analysis) [18]. A “personalized” random forest approach to distinguish candidemia from bacteremia in internal medicine wards achieved an AUC of 0.87, sensitivity of 0.84, and specificity of 0.91 in the reported test set [19]. The CanDETEC random forest model, developed in a hematologic malignancy population, yielded an AUC of 0.889, sensitivity of 0.901, specificity of 0.722, PPV of 0.449, and NPV of 0.967 in the validation cohort [20]. An XGBoost-based model applied to ICU patients with new-onset SIRS showed an AUC of 0.924, sensitivity of 0.84, and specificity of 0.89 in an internal test set (also used in our meta-analysis); the very high NPV (~0.996) and low PPV (~0.13) were consistent with the low prevalence of candidemia in the cohort [21]. Two additional studies developed candidemia prediction models but were not included in our meta-analysis due to non-harmonizable performance reporting (i.e., lack of sensitivity/specificity at a clearly defined threshold to construct 2 × 2 contingency data) [22,23]. These studies reported internal validation only, with a random forest model in ICU patients (AUC ~0.81) and a penalized logistic regression model focusing on *Candida auris* candidemia (AUC ~0.89), respectively [22,23].

One study focused on invasive candidiasis in the intra-abdominal setting. This prospective study developed a machine-learning-driven nomogram combining clinical features and immunological markers (including lymphocyte subtypes) to predict intra-abdominal candidiasis in ICU patients with severe intra-abdominal infections. The final model achieved an AUC of 0.822 with sensitivity 0.798 and specificity 0.707 in its validation data. Notably, this was the only study among the included ones that was prospective in design, and it did not address candidemia but rather deep-seated candidiasis [26].

### 3.5. Meta-Analysis

Five of the seven candidemia-focused studies had sufficient data (or provided data enabling the reconstruction of confusion matrices) to be included in the quantitative meta-analysis [17,18,19,20,21]. Together, these five studies encompassed a pooled total of 14,620 patients or infection episodes, of which 1215 were candidemia cases. Table 4 summarizes the key performance metrics and cohort characteristics of these studies. Study-level 2 × 2 contingency tables (TP, FP, TN, FN) used to derive sensitivity and specificity for the quantitative synthesis are provided in the Supplementary Materials (Table S2). Based on the bivariate random-effects model, the pooled sensitivity of the ML models for identifying candidemia was 0.813 (95% confidence interval (CI) 0.729–0.876), and the pooled specificity was 0.816 (95% CI 0.684–0.901). Between-study heterogeneity was substantial (bivariate I^2^ = 86.6%), with between-study variance on the logit scale of 0.25 for sensitivity and 0.61 for specificity. This corresponds to a summary diagnostic odds ratio (DOR) of approximately 19.3 (95% CI 8.1–45.7), indicating that these AI/ML models were about 19 times more likely to make a correct positive prediction in a patient with candidemia than a false-positive prediction in a non-candidemia patient. In practical terms, at the study-reported operating thresholds, the models achieved a moderate balance of sensitivity and specificity for early candidemia prediction.

**Figure 2:**
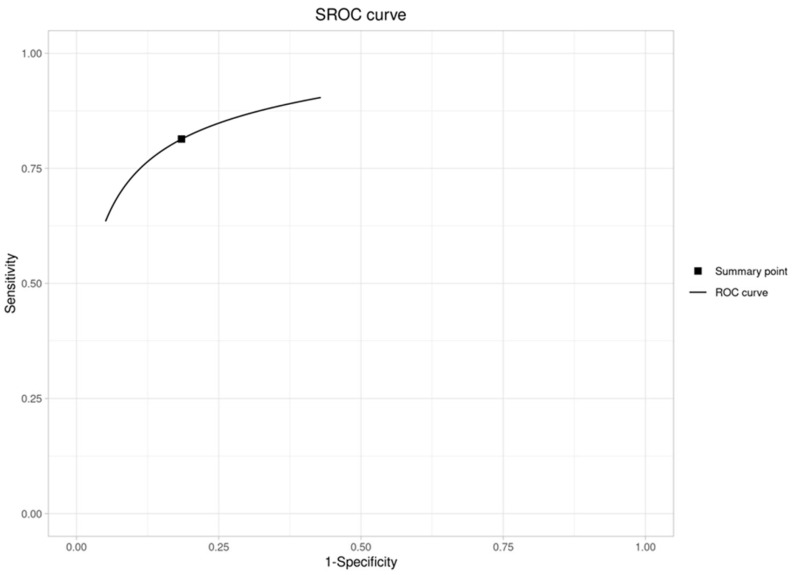
SROC curve.

**Figure 3:**
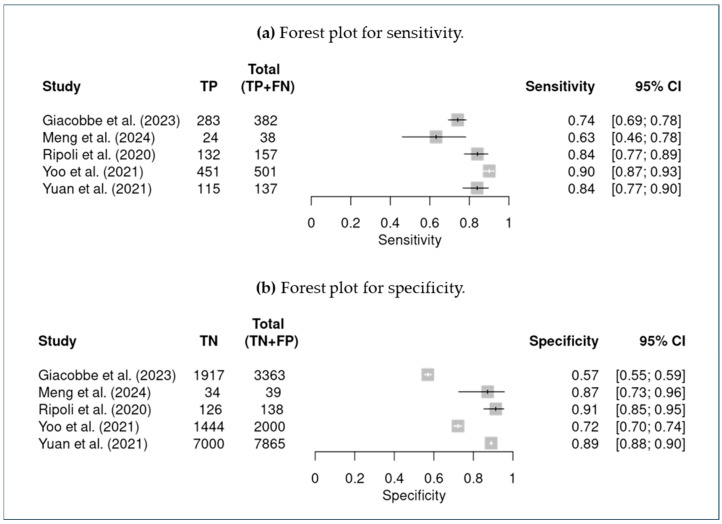
Forest plot: (**a**) sensitivity; and (**b**) specificity. Giacobbe et al. (2023) [17]; Meng et al. (2024) [18]; Ripoli et al. (2020) [19]; Yoo et al. (2021) [20]; Yuan et al. (2021) [21].

In addition to sensitivity and specificity, we derived secondary diagnostic parameters from the meta-analysis. The summary positive likelihood ratio (LR+) was approximately 4.42 (95% CI ~2.45–7.99), and the summary negative likelihood ratio (LR−) was 0.23 (95% CI ~0.15–0.35). These values suggest that a positive result from an AI model (above the alert threshold) would increase the odds of actual candidemia by about fourfold, while a negative result would reduce the odds to about one-fourth, in the contexts studied. Figure 2 illustrates the summary receiver operating characteristic curve (SROC curve) for the pooled analysis, showing the trade-off between sensitivity and specificity across the included studies. Table 5 recompiles diagnostic parameters. Forest plot graphics for sensitivity and specificity are available in Figure 3.

Reporting bias/publication bias was not formally assessed due to the limited number of studies contributing to the quantitative synthesis and the known limitations of publication-bias tests in diagnostic accuracy meta-analyses.

Overall, the pooled results support a moderate-to-good discriminative performance of current AI/ML models for early candidemia detection in high-risk settings. However, there was notable between-study heterogeneity in terms of patient populations, feature sets, and validation approaches, which is reflected in the width of the confidence intervals and the variability of PPV/NPV across studies. This heterogeneity cautions against over-generalization of the pooled point estimates to all clinical scenarios.

## 4. Discussion

Our systematic review synthesizes current evidence on AI/ML approaches for the early identification and risk prediction of invasive *Candida* infections, with candidemia being the primary focus of quantitative analysis. Across eight included studies, models were typically built using routinely collected clinical and laboratory variables and applied in heterogeneous hospital populations. In our meta-analysis of five studies focused on candidemia, the models demonstrated moderate diagnostic accuracy, with pooled sensitivity and specificity of approximately 81% and 82%, respectively. This finding supports the potential of AI-assisted risk stratification to complement conventional diagnostic workflows for invasive candidiasis. At the same time, the evidence base is constrained by methodological heterogeneity, limited external validation, and frequent risks of bias. These limitations emphasize the need for better-designed and more transparently reported studies before AI/ML models can be implemented at scale in clinical practice.

### 4.1. Interpretation of the Principal Findings

The pooled sensitivity and specificity estimates suggest that, at operating thresholds used in these studies, ML models can meaningfully discriminate patients with candidemia from those without candidemia in certain high-risk settings. Clinically, this is relevant because the early recognition of candidemia is difficult and time-sensitive (delayed or missed diagnoses can lead to poor outcomes) while over-treatment with empiric antifungals can cause toxicity and promote resistance. A common theme across studies was that the models tended to have high negative predictive values. In a low-prevalence scenario, a high NPV means the model may serve well as a rule-out tool: if the model predicts a patient to be low-risk, clinicians might feel more confident withholding empiric antifungal therapy or discontinuing it early. On the other hand, positive predictive values were often modest. A low PPV indicates that many “high-risk” alerts might be false positives, which is not unexpected given baseline infection rates and, in some studies, the use of enriched cohorts for model training. Therefore, current AI models might be better suited for prioritization tasks (e.g., identifying which patients warrant enhanced monitoring, targeted diagnostics, or preemptive therapy) rather than functioning as standalone diagnostic tests that definitively “rule in” candidemia. In practice, an ML prediction should augment, not replace, clinical judgment and microbiological confirmation.

### 4.2. Why Results Vary Across Studies

We observed several likely drivers of between-study variability in model performance and generalizability, including

1. Population and setting differences: Some models were developed in ICU populations, others in general internal medicine wards, oncology units, or highly selected patient groups. These differences alter the baseline risk of candidemia, the distribution of predictors, and the practical thresholds for triggering interventions. A model trained to distinguish candidemia from bacteremia in ICU patients, for example, is not directly equivalent to one aiming to detect candidemia among all febrile patients hospital-wide. When models are applied outside their development context, performance can be inflated or deflated unpredictably.
2. Outcome definition and comparator group: Included candidemia studies varied in their outcome definitions and comparison groups. Several models specifically contrasted candidemia cases against bacteremia cases or other infected controls, while others used broader control groups (e.g., any non-candidemia patient). A model trained to differentiate candidemia from bacteremia addresses a different clinical question than one that identifies candidemia among all ICU admissions with sepsis. Such differences affect how results should be interpreted; a model’s utility is linked to the context and question it was built for.
3. Timing of prediction (“time-zero”) and predictor availability: A key consideration for any “early diagnosis” model is the time-point at which it is intended to be used. Some studies set time-zero at ICU admission, others at the moment of blood culture collection or onset of sepsis. Predictors must be available by that time to be truly useful. If a model relies on cumulative exposures (“days on antibiotics” or persistent fevers), it may inadvertently incorporate future information (look-ahead bias) if not carefully designed. We noted that inconsistent or unclear definitions of time-zero and the inclusion of time-dependent variables were potential issues in several studies, which could lead to the overestimation of performance in real prospective deployment.
4. Validation approach: Truly independent external validation was rare. Most studies only performed internal validation (random hold-out sets, cross-validation, or temporal splits within the same center). While these methods mitigate overfitting to some extent, they do not guarantee that a model will transport well to a different hospital or patient population. EHR-derived models can pick up on institution-specific patterns (e.g., local prescribing habits or lab usage) that may not hold elsewhere. The one study in our review that did include external validation [21], with a multi-center ICU cohort) showed some drop in performance between development and validation, underscoring the importance of this step. In general, the lack of external validation in most studies means the field has yet to truly prove that these AI tools work broadly, outside of their development environments.

### 4.3. Methodological Quality and Certainty of Evidence

The overall risk of bias for the included studies was concerning: using PROBAST, all studies were rated as having either moderate or high risk of bias. Common issues were identified in the participant selection domain (many studies used retrospective cohorts or case-control sampling that can introduce bias), predictor domain (unclear if predictors were measured without knowledge of the outcome and truly available at prediction time), and analysis domain (some studies did not report how they handled missing data or whether model overfitting was addressed, etc.) [27]. These biases mean that reported performance might be overly optimistic. In terms of applicability, many models were developed in niche populations (e.g., a single ICU or a specific subset of patients) which limits their direct applicability to other settings [28].

Correspondingly, when we applied GRADE to evaluate the certainty of the evidence, we found the evidence supporting AI/ML model accuracy for early candidemia to be of low to very low certainty. In practical terms, this means we have low confidence that the pooled sensitivity and specificity reflect the true values these models would achieve in routine practice across diverse settings. The certainty was downgraded primarily due to the risk-of-bias issues and indirectness (each study addresses a slightly different question, population, or comparator) [28]. Inconsistency was somewhat present (there was moderate heterogeneity in the results, though not extreme given the overlapping CIs), and imprecision was a factor for some metrics. While this does not negate the potential usefulness of AI models, it highlights that current studies likely represent best-case scenarios within controlled contexts. Real-world performance and impact on patient outcomes remain largely unproven at this stage.

### 4.4. Relation to Prior Literature and Clinical Implications

Our findings align with prior reviews and expert commentary indicating that predictive models for invasive candidiasis often suffer from limited generalizability and a lack of rigorous validation [16]. A systematic review of clinical predictive models (not limited to AI/ML) for invasive candidiasis concluded that many models had overly optimistic performance that might not translate well to everyday practice [16]. The present review builds upon prior literature by focusing specifically on modern AI/ML approaches and by providing a quantitative synthesis of diagnostic accuracy for candidemia. By pooling data from multiple studies, we offer a benchmark for what current ML models can achieve in terms of sensitivity and specificity.

From a clinical perspective, AI-based tools for fungal infection could be most useful if integrated into existing antimicrobial stewardship and diagnostic pathways. For example, a risk prediction model could automatically alert clinicians when a patient’s risk of candidemia exceeds a certain threshold, prompting confirmatory testing (like beta-D-glucan or PCR assays) or early antifungal treatment parallel with cultures. Conversely, a low-risk prediction might support a decision to withhold empiric antifungals in a febrile patient pending more data. Importantly, none of these models are meant to replace blood cultures or other definitive diagnostics but rather to act as decision-support. Implementation should emphasize transparency (so clinicians understand why a model is flagging a patient), calibration (so the predicted risk corresponds to actual probability), and appropriate thresholds that align with clinical decision points. Additionally, any model adopted should undergo local validation; even the best-performing published model might perform differently at a new institution due to case-mix and practice differences.

### 4.5. Strengths and Limitations

This review has several strengths. We conducted a comprehensive literature search across multiple databases with a peer-reviewed strategy (detailed in Supplementary File S1) and followed a registered protocol and PRISMA 2020 reporting standards. We focused our primary analysis on a clearly defined outcome (candidemia) and used established meta-analytic methods for diagnostic accuracy. We explicitly addressed the issue of overlapping patient cohorts by identifying and excluding duplicate analyses, thereby ensuring that each dataset contributed only once to our synthesis. We also performed standardized risk-of-bias and certainty assessments (PROBAST and GRADE), which provide a deeper understanding of the quality of evidence beyond just the performance numbers.

Nonetheless, there are important limitations. The number of studies we identified was relatively small (only eight met inclusion criteria), and only five could be quantitatively pooled. This limited our ability to explore heterogeneity or perform meaningful subgroup analyses (e.g., by ICU vs non-ICU setting). Furthermore, the certainty of the evidence was rated as low to very low according to GRADE criteria, mainly due to the observational nature of the studies, most of which were retrospective (7 out of 8). Thus, the heterogeneity in study designs, patient populations, and model features was substantial, which complicates any attempt to generalize findings. Our meta-analysis had to assume that, despite differences, pooling was justified to estimate an “average” performance; this assumption may not hold if some models addressed fundamentally different questions. Also, several studies did not report all metrics of interest (for instance, not all provided sensitivity/specificity at a given threshold, or calibration statistics), which led to their exclusion from quantitative synthesis and could introduce reporting bias. Finally, because of the rapid evolution of the AI field, there may be recent or ongoing studies (e.g., conference papers, preprints) that were not captured in our search or that have emerged since our search date, highlighting the need for continual updates in this domain.

### 4.6. Future Directions

Building on the findings of this review, we recommend several directions for future research on AI in invasive fungal infection diagnosis:

1. Clinical timing (“time-zero”) alignment: Future models should clearly define the clinical decision point at which they are meant to be used (e.g., on ICU admission, on onset of sepsis, at the time of culture draw) and ensure that all predictors are available by that point. This will enhance clinical applicability and avoid biases from using information that would not actually be known in real time.
2. Transparent feature Engineering and Avoidance of Data Leakage: Researchers should report how predictors are selected and processed and take care to avoid inadvertently using post-outcome or future data in model training. Techniques like proper time-splitting of data and using only baseline features can help ensure models do not overestimate performance.
3. Comprehensive reporting of performance (discrimination and calibration): Besides AUC and sensitivity/specificity, studies should report calibration (how well predicted risks match observed outcomes) and provide confidence intervals for all metrics. This allows for a better assessment of clinical utility. Open sharing of code and data (when possible) can also facilitate external validation and trust in the models.
4. External validation across diverse settings: It is crucial to test promising models on truly external datasets from different hospitals or patient populations. Such validation will reveal how generalizable a model is and identify any needed adjustments. Ideally, multi-center collaborations or consortia should be formed to evaluate models across healthcare systems and geographies, especially for rare outcomes like invasive candidiasis.
5. Prospective impact studies: Ultimately, to justify clinical adoption, we need studies that implement these AI models in real clinical workflows and measure outcomes. For example, a randomized trial or stepped-wedge study could evaluate whether an AI-driven alert system for candidemia risk actually leads to earlier antifungal therapy, reduced mortality, or improved antifungal stewardship (e.g., less unnecessary therapy) compared to standard care. Such impact studies will help determine if the theoretical benefits of AI translate into tangible patient benefits and more efficient care.

## 5. Conclusions

AI and machine-learning models for the early identification of candidemia (and other invasive *Candida* infections) demonstrate moderate diagnostic accuracy in published studies to date. In particular, these tools can achieve pooled sensitivity and specificity in the low 80% range for candidemia, suggesting they have potential to aid in timely diagnosis and treatment decisions. Despite promising performance, the current evidence is limited by substantial heterogeneity between studies, their retrospective observational design, a lack of widespread external validation, and common risks of bias in model development. Therefore, while AI/ML models may be most valuable as adjunctive decision-support systems within diagnostic and antifungal stewardship pathways (for example, helping to rule out infection in low-risk cases or prompt early investigation in high-risk cases), they cannot yet replace standard diagnostic approaches. Further robust validation, ideally in prospective, multicenter settings, along with transparent reporting and demonstration of clinical impact, is needed before these AI-driven models can be recommended for routine clinical adoption.

## Figures and Tables

**Table 1:** Studies’ characteristics.

[Ref.]	Study Site	Country	ICU	Infection Type	Study Design	AI Model
[23]	Emory University Hospital (Atlanta)	USA	Yes	Candidemia	Retrospective case-control study	Random Forest
[22]	Hospital La Fe (Valencia)	Spain	Yes	Candidemia	Retrospective cohort study	Logistic regression
[17]	IRCCS Ospedale Policlinico San Martino (Génova)	Italy	No	Candidemia	Retrospective study	Logistic regression
[18]	Jining No.1 People’s Hospital and Tengzhou Central People’s Hospital	China	Yes	Candidemia	Retrospective study	Random Forest
[19]	Multiple internal medicine wards (Pisa, Firenze and Verona)	Italy	No	Candidemia	Retrospective study	Random Forest
[20]	Samsung Medical Center (Seúl)	South Korea	No	Candidemia	Retrospective study	Random Forest
[21]	Peking Union Medical College Hospital; Affiliated Hospital of Qingdao Univ; First Affiliated Hospital of Fujian Medical Univ.	China	Yes	Candidemia	Retrospective study	XGBoost
[26]	Peking Union Medical College Hospital (Beijing)	China	Yes	Intra-abdominal candidiasis	Prospective cohort study	Random Forest

**Table 2:** PROBAST risk-of-bias.

[Ref.]	Participants (RoB ^1^/App ^2^)	Predictors (RoB/App)	Outcome (RoB/App)	Analysis (RoB)	Overall
[23]	high/high	high/high	low/moderate	high	high
[22]	moderate/high	moderate–high/high	low/moderate	moderate	moderate–high
[17]	moderate/moderate	low–moderate/moderate	low/low	moderate	moderate
[18]	high/moderate–high	moderate/moderate	low/moderate	high	high
[19]	high/moderate	moderate–high/moderate	low/low	moderate	moderate–high
[20]	moderate/high	moderate/moderate	low/moderate	moderate	moderate
[21]	low–moderate/moderate	moderate–high/moderate	low/low	moderate–high	moderate–high
[26]	low/moderate	probably low/moderate	low/low	moderate	moderate

^1^ RoB: Risk of Bias. ^2^ App: Applicability.

**Table 3:** GRADE certainty assessment.

[Ref.]	Target Condition/Outcome	Risk of Bias	Indirectness	Imprecision	Preliminary Certainty
[23]	Candidemia	very serious	serious	very serious	very low
[22]	Candidemia	serious	very serious	serious	very low
[17]	Candidemia	serious	minor–serious	not serious	low
[18]	Candidemia	very serious	minor–serious	serious	very low
[19]	Candidemia	serious	minor–serious	not serious	low
[20]	Candidemia	serious	serious	not serious	low
[21]	Candidemia	serious	minor–not serious	not serious	low
[26]	Intra-abdominal candidiasis	serious	serious	not serious–minor	low

**Table 4:** Performance metrics.

[Ref.]	n_Total	n_Events	n_Controls	AUC	Sensitivity	Specificity
[17]	3745 *	382	3363	NR	0.74	0.57
[18]	77 **	38	39	0.870	0.63	0.87
[19]	295	157	138	0.87	0.84	0.91
[20]	2501	501	2000	0.889	0.901	0.722
[21]	8002	137	7865	0.924	0.84	0.89

* Approximated “n” (30% for 12,483 episodes). ** In Meng, we use the external cohort for meta-analysis.

**Table 5:** Diagnostic parameters.

Variable	Estimate	95% LCI	95% UCI
**Sensitivity**	0.813	0.729	0.876
**Specificity**	0.816	0.684	0.901
**DOR**	19.287	8.133	45.736
**LR+**	4.419	2.445	7.985
**LR−**	0.229	0.152	0.346
**FPR**	0.184	0.099	0.316

## Data Availability

All data supporting the findings are derived from published studies included in this systematic review. Extracted and derived datasets are provided in this manuscript and Supplementary Materials.

## Supplementary Materials

The following supporting information can be downloaded at https://www.mdpi.com/article/10.3390/jof12020138/s1, Supplementary File S1: Search strategy. Table S1: Patient characteristics and predictive variables; Table S2: Study-level 2 × 2 contingency tables; Table S3: PRISMA 2020 abstract checklist; Table S4: Prisma 2020 checklist.

## Abbreviations

The following abbreviations are used in this manuscript:

AI	Artificial Intelligence
AKI	Acute kidney injury
AUC	Area Under the Receiver Operating Characteristic Curve
CI	Confidence Interval
CVC	Central venous catheter
DOR	Diagnostic Odds Ratio
EHR	Electronic Health Record
GI	Gastrointestinal
GRADE	Grading of Recommendations Assessment, Development and Evaluation
Hb	Hemoglobin
Hct	Hematocrit
HSCT	Hematopoietic Stem Cell Transplantation
IAC	Intra-abdominal candidiasis
ICU	Intensive Care Unit
IFI	Invasive Fungal Infection
LR−	Negative Likelihood Ratio
LR+	Positive Likelihood Ratio
LVAD	Left ventricular assist device
ML	Machine Learning
NPV	Negative Predictive Value
PCT	Procalcitonin
PICC	Peripherally inserted central catheter
PLT	Platelet count
PPV	Positive Predictive Value
PRISMA	Preferred Reporting Items for Systematic Reviews and Meta-Analyses
PROBAST	Prediction Model Risk of Bias Assessment Tool
ROC	Receiver Operating Characteristic
RR var (7d)	7-day variance of respiratory rate
RRT	Renal replacement therapy
SBP var (7d)	7-day variance of systolic blood pressure
SIRS	Systemic inflammatory response syndrome
SROC	Summary Receiver Operating Characteristic
TBil	Total bilirubin
TPN	Total parenteral nutrition
WBC	White blood cell count
