# Supplementary material for: Can Artificial Intelligence Optimize the Early Diagnosis of Invasive Candidiasis? A Systematic Review and Meta-Analysis
Source: J Fungi (Basel). 2026 Feb 13;12(2):138. doi: 10.3390/jof12020138 (PMC12942343; doi:10.3390/jof12020138)
Supplement: Supplementary file 1 [file jof-12-00138-s001.zip › Supplementary File S1. Search strategy.pdf]

Supplementary File S1. Search strategy (**Global search date:** December 22, 2025).

PubMed/MEDLINE

(("Artificial Intelligence"[MeSH] OR "Machine Learning"[MeSH] OR "Neural Networks, Computer"[MeSH]
OR "Deep Learning"[MeSH] OR artificial intelligence[tiab] OR machine learning[tiab]
OR deep learning[tiab] OR neural network*[tiab] OR random forest*[tiab] OR xgboost[tiab]
OR predictive model*[tiab] OR automated diagnosis[tiab] OR algorithm*[tiab]))
AND
(("Candidemia"[MeSH] OR "Candidiasis, Invasive"[MeSH]
OR candidemia[tiab] OR candidaemia[tiab] OR "invasive candidiasis"[tiab]
OR "invasive candidiasis"[tiab] OR "Candida bloodstream infection"[tiab]
OR fungemia[tiab] OR "Candida sepsis"[tiab]
OR ("Candida"[tiab] AND (invasive[tiab] OR disseminated[tiab] OR deep-seated[tiab] OR "organ"[tiab]))))
AND
(("Immunocompromised Host"[MeSH] OR "Neutropenia"[MeSH]
OR "Hematopoietic Stem Cell Transplantation"[MeSH]
OR immunocompromised[tiab] OR immunosuppression[tiab] OR neutropenia[tiab]
OR neutropenic[tiab] OR stem cell transplant*[tiab] OR HSCT[tiab]
OR transplant recipient*[tiab] OR ICU[tiab] OR HIV[tiab]))
AND
(("Early Diagnosis"[MeSH] OR diagnosis[tiab] OR detection[tiab] OR prediction[tiab]
OR diagnostic accuracy[tiab] OR biomarker*[tiab]))

- Filters: Publication date 2015–2025.

Embase (Embase.com)

(('artificial intelligence'/exp OR 'machine learning'/exp OR 'deep learning'/exp OR 'neural network'/exp
OR (artificial intelligence OR machine learning OR deep learning OR neural network* OR random forest*
OR xgboost OR predictive model* OR automated diagnosis OR algorithm*):ti,ab,kw))
AND
(('candidemia'/exp OR 'invasive candidiasis'/exp OR 'candida infection'/exp
OR (candidemia OR candidaemia OR "invasive candidiasis" OR "candida bloodstream infection"
OR fungemia OR "candida sepsis" OR (candida AND (invasive OR disseminated OR deep-seated OR organ))):ti,ab,kw))
AND
(('immunocompromised host'/exp OR 'neutropenia'/exp OR 'stem cell transplantation'/exp
OR 'organ transplantation'/exp OR (immunocompromised OR immunosuppression OR neutropenia OR neutropenic
OR stem cell transplant* OR HSCT OR transplant recipient* OR ICU OR HIV):ti,ab,kw))
AND

(('diagnosis'/exp OR 'early diagnosis'/exp OR 'prediction'/exp
OR (diagnosis OR detection OR prediction OR diagnostic accuracy OR biomarker*):ti,ab,kw))

- Filters: Publication date 2015–2025.

Web of Science Core Collection

TS=(("artificial intelligence" OR "machine learning" OR "deep learning" OR "neural network*"
OR "convolutional neural network*" OR "random forest*" OR "decision tree*"
OR "support vector machine*" OR xgboost OR "gradient boosting"
OR "predictive model*" OR "prediction model*" OR "automated diagnosis"
OR "computer aided diagnosis" OR algorithm*))
AND
TS=((candidemia OR candidaemia OR "invasive candidiasis" OR "candida bloodstream infection"
OR fungemia OR "candida sepsis"
OR (candida AND (invasive OR disseminated OR "deep-seated" OR "deep seated" OR organ))))
AND
TS=((immunocompromised OR immunosuppression OR immunosuppressed OR neutropenia OR neutropenic
OR "febrile neutropenia" OR "hematologic malignancies" OR "hematological malignancy"
OR "stem cell transplant*" OR HSCT OR "bone marrow transplant*" OR "solid organ transplant*"
OR SOT OR "transplant recipient*" OR ICU OR "intensive care unit" OR "critically ill" OR HIV OR AIDS))
AND
TS=((diagnosis OR diagnostic OR "early diagnosis" OR detection OR prediction OR predictive
OR "risk stratification" OR "diagnostic accuracy" OR sensitivity OR specificity OR biomarker*))

- Filters: Publication date 2015–2025; Document types ≠ Letter, Meeting Abstract, Book Chapter, Editorial.

Scopus

TITLE-ABS-KEY((("artificial intelligence" OR "machine learning" OR "deep learning" OR "neural network*"
OR "convolutional neural network*" OR "random forest*" OR "decision tree*"
OR "support vector machine*" OR xgboost OR "gradient boosting" OR "predictive model*"
OR "prediction model*" OR "automated diagnosis" OR "computer aided diagnosis" OR algorithm*))
AND
((candidemia OR candidaemia OR "invasive candidiasis" OR "candida bloodstream infection"
OR fungemia OR "candida sepsis"

OR (candida AND (invasive OR disseminated OR "deep-seated" OR "deep seated" OR organ))))
AND
((immunocompromised OR immunosuppression OR immunosuppressed OR neutropenia OR neutropenic
OR "febrile neutropenia" OR "hematologic malignancies" OR "hematological malignancy"
OR "stem cell transplant*" OR HSCT OR "bone marrow transplant*" OR "solid organ transplant*"
OR SOT OR "transplant recipient*" OR ICU OR "intensive care unit" OR "critically ill" OR HIV OR AIDS))
AND
((diagnosis OR diagnostic OR "early diagnosis" OR detection OR prediction OR predictive
OR "risk stratification" OR "diagnostic accuracy" OR sensitivity OR specificity OR biomarker*)))

- Filters: Publication date 2015–2025; Document type: Article, Review.

IEEE Xplore Digital Library

("All Metadata":"artificial intelligence" OR "All Metadata":"machine learning"
OR "All Metadata":"deep learning" OR "All Metadata":"neural network"
OR "All Metadata":"convolutional neural network" OR "All Metadata":"random forest"
OR "All Metadata":"predictive model")
AND
("All Metadata":"candidemia" OR "All Metadata":"candidaemia"
OR "All Metadata":"invasive candidiasis" OR "All Metadata":"candida bloodstream infection"
OR "All Metadata":"fungemia" OR "All Metadata":"candida sepsis"
OR ("All Metadata":"candida" AND ("All Metadata":"invasive" OR "All Metadata":"disseminated" OR "All Metadata":"organ")))
AND
("All Metadata":"diagnosis" OR "All Metadata":"detection"
OR "All Metadata":"prediction" OR "All Metadata":"classification")

Cochrane Library

#1 MeSH descriptor: [Artificial Intelligence] explode all trees
#2 MeSH descriptor: [Machine Learning] explode all trees
#3 MeSH descriptor: [Neural Networks, Computer] explode all trees
#4 MeSH descriptor: [Deep Learning] explode all trees
#5 (artificial intelligence OR machine learning OR deep learning OR neural network*
OR random forest OR xgboost OR predictive model* OR automated diagnosis OR algorithm*):ti,ab,kw
#6 {OR #1-#5}

#7 MeSH descriptor: [Candidemia] explode all trees
#8 MeSH descriptor: [Candidiasis] explode all trees
#9 ("invasive candidiasis" OR candidemia OR candidaemia OR "candida bloodstream infection"

OR fungemia OR "candida sepsis" OR (candida AND (invasive OR disseminated OR "deep-seated" OR organ))):ti,ab,kw
#10 {OR #7-#9}

#11 MeSH descriptor: [Immunocompromised Host] explode all trees
#12 MeSH descriptor: [Neutropenia] explode all trees
#13 MeSH descriptor: [Hematopoietic Stem Cell Transplantation] explode all trees
#14 (immunocompromised OR immunosuppression OR neutropenia
OR hematologic malignancies OR stem cell transplant* OR HSCT
OR transplant recipient* OR ICU OR HIV):ti,ab,kw
#15 {OR #11-#14}

#16 #6 AND #10 AND #15 with Publication Year from 2015 to 2025
