# Supplementary material for: Can Artificial Intelligence Optimize the Early Diagnosis of Invasive Candidiasis? A Systematic Review and Meta-Analysis
Source: J Fungi (Basel). 2026 Feb 13;12(2):138. doi: 10.3390/jof12020138 (PMC12942343; doi:10.3390/jof12020138)
Supplement: Supplementary file 1 [file jof-12-00138-s001.zip › Table S1. Patient characteristics and predictive variables v2.pdf]

Table S1. Patient characteristics and predictive variables

[Ref.]	Patient characteristics	Variables
**[23]**	Left ventricular assist device (LVAD) recipients (single-center cohort).	Duration of mechanical ventilation, renal replacement therapy (RRT), total parenteral nutrition (TPN)
**[22]**	Critically ill ICU population evaluated for *Candida auris* candidemia.	TPN, previous surgery, sepsis, previous exposure to antifungal therapy, arterial catheter, central venous catheter (CVC), advanced chronic kidney disease, multifocal colonization
**[17]**	Large episode-level dataset (candidemia vs bacteremia episodes) using routinely extracted labs and *Candida* colonization data.	(1,3)-β-D-glucan, procalcitonin (PCT), eosinophil count, platelet count (PLT), neutrophil count, Hct, uric acid, monocyte count, Hb, urea, albumin, lymphocyte count, WBC, prothrombin activity
**[18]**	ICU patients: candidemia vs bacteremia; internal split plus external validation cohort.	Total bilirubin (TBil), Age, PLT, Hb, CVC, lymphocyte count, duration of ICU stay, neutrophil count, monocyte count, antibiotic therapy, PCT, C-reactive protein, WBC, immunosuppressive therapy, TPN, abdominal surgery, chemotherapy, solid cancer
**[19]**	Hospitalized adult population (real-world cohort) used to build a personalized ML approach.	Age, fever, previous antibiotic therapy, in-hospital antibiotic therapy, in-hospital immunosuppressive therapy, in-hospital corticosteroids therapy, in-hospital microbiome highly impacting antimicrobials, TPN, PICC, urinary catheter, nasogastric tube, Dementia, cerebrovascular disease, peripheral vascular disease, Charlson comorbidity index/score, Concomitant infection, Admission from home
**[20]**	Hospitalized population with blood-culture based case–control design; models trained using auto-extractable EHR variables.	blood urea nitrogen, RR var (7d), TBil, SBP var (7d), body weight
**[21]**	ICU patients with new-onset SIRS; multi-hospital retrospective dataset.	Fungal colonization, diabetes mellitus, AKI, TPN days, RRT
**[26]**	Elderly cohort evaluated for intra-abdominal candidiasis (IAC), including immune/lymphocyte subset measurements.	GI perforation, RRT, T-cell count, CD28+CD8+ T cells, CD38+CD8+ T cells
