# Supplementary material for: Can Artificial Intelligence Optimize the Early Diagnosis of Invasive Candidiasis? A Systematic Review and Meta-Analysis
Source: J Fungi (Basel). 2026 Feb 13;12(2):138. doi: 10.3390/jof12020138 (PMC12942343; doi:10.3390/jof12020138)
Supplement: Supplementary file 1 [file jof-12-00138-s001.zip › Table S2. Study-level 2×2 contingency tables v2.pdf]

Table S2. Study-level 2×2 contingency tables

[Ref]	TP	FN	FP	TN
[17]	283	99	1446	1917
[18]	24	14	5	34
[19]	132	25	12	126
[20]	451	50	556	1444
[21]	115	22	865	7000
